# Editorial: Dynamic of Transmissible Diseases: Integrative and Transdisciplinary Approaches

**DOI:** 10.3389/fpubh.2022.862069

**Published:** 2022-02-24

**Authors:** Roger Frutos, Laurent Gavotte, Jordi Serra-Cobo, Christian A. Devaux

**Affiliations:** ^1^Cirad, UMR 17, InterTryp, Montpellier, France; ^2^Espace-Dev, Université de Montpellier, Montpellier, France; ^3^Department of Evolutionary Biology, Ecology and Environmental Sciences, Faculty of Biology, University of Barcelona, Biodiversity Research Institute (IRBIO), Barcelona, Spain; ^4^MEPHI, Aix-Marseille Université, IRD, AP-HM, IHU-Méditerranée Infection, Marseille, France; ^5^CNRS, Marseille, France

**Keywords:** infectious diseases, integrative, transdisciplinary, scientific literature, epidemic, pandemic

The topic “*Dynamic of Transmissible Diseases: Integrative and Transdisciplinary Approaches*” proposed jointly by Frontiers in Medicine and Frontiers in Public Health has attracted a total of six publications. This might seem little, and yes somehow it is, but it is also a success because the topic has achieved its objective of opening the way to works which are usually not published in front line journals. Medicine and public health, but also biology and science at large have become divided into highly specialized fields. This is necessary in order to cope with the progress in technology and knowledge. However, doing so, these highly specialized topics are cutting links with the biological reality. Life is based on a multitude interactions and reactions at different levels of complexity. Life is also fractal. An infectious disease is only the last occurrence in a series of events. As a disease, it is the description of a panel of specific symptoms but these symptoms are only the consequences of the life cycle of a living organism on another living organisms. Although very important, medicine is only addressing a tiny part of the whole, complex and multilevel interactive process leading to an infectious disease. Infectious microorganisms are also taking advantage of, and sometimes changing the ecology and ethology of the host to facilitate propagation. The ecology and ethology of the human species correspond to what is known as the human society with societal rules having replaced ecological rules. Therefore, the emergence of an infectious disease is a complex process influenced by ecology (host-pathogen interactions), sociology (host behavior), physiology (host defense), biochemistry (molecular dialogue between host and pathogen) and medicine (host reaction to the pathogen). Highly specialized discipline cannot provide by itself the comprehensive view required to understand the whole process. Scientific journals being also highly specialized, the published articles, although scientifically and technically good and relevant, only reflect a small part of the whole process. In order to be described processes must be cut down into small entities (the reductionist approach) but in order to be understood processes must be considered in a comprehensive way (the integrative approach). Since the current scientific literature is mostly directed toward the description of phenomena, opening the literature space to more interdisciplinary and integrative works is necessary and unavoidable. This what we have tried to do with this topic. It is only a first step. Three of the six articles published in the topic are modeling studies, i.e., “*Epidemiological Characteristics and Transmissibility of Human Immunodeficiency Virus in Nanning City, China, 2001–2020*” by Lin et al., “*Meteorological Factors and the Transmissibility of Hand, Foot, and Mouth Disease in Xiamen City, China*” by Xu et al., and “*Exponential Damping: The Key to Successful Containment of COVID-19*” by Zhang et al. Modeling is by nature a multifactorial approach leading to a rather comprehensive understanding. This is what has been achieved in these three articles dealing with three different diseases, i.e., AIDS, HFMD and COVID-19. Two other articles of the series are reviews, i.e., “*Coronavirus Disease (COVID-19): Comprehensive Review of Clinical Presentation*” by Mehta et al. and “*Aerosol Transmission of SARS-CoV-2: Physical Principles and Implications*” by Jarvis, which are covering in a comprehensive way, different aspects of the COVID-19 pandemic with the aim of better understanding the various implications. The last article of the series, “*Exclusivity of Cultural Practices Within Emerging Disease Outbreak Responses in Developing Nations Leads to Detrimental Outcomes*” by Lal, is a perspective article addressing in a comprehensive way cultural, cross-cultural and societal response to epidemics in order to reach a global perspective. Pandemics are by definition international and cross-cultural aspects are thus essential. There is hardly anything more societal than an epidemic or a pandemic. We, as a scientific community, tend to ignore that and focus on highly specific and technical aspects. However, although important, these technical aspects cannot alone explain the emergence and the propagation of an infectious disease. Providing space for articles covering integrative and comprehensive approaches is essential to consider the problem of infectious diseases from a global perspective and to be able to perceive the whole process. The COVID-19 crisis has clearly demonstrated that despite the enormous number of articles published on this disease over the last 2 years, very little progress has been made in the understanding the dynamic of the disease and on its control. The blame is on too much specialization and compartmentalization of the research and not enough integration. More dedicated sections and dedicated journals should be devoted to integrative and transdisciplinary approaches. It is the key for a better understanding, better management and better prevention.

## Author Contributions

All authors listed have made a substantial, direct, and intellectual contribution to the work and approved it for publication.

## Conflict of Interest

The authors declare that the research was conducted in the absence of any commercial or financial relationships that could be construed as a potential conflict of interest.

## Publisher's Note

All claims expressed in this article are solely those of the authors and do not necessarily represent those of their affiliated organizations, or those of the publisher, the editors and the reviewers. Any product that may be evaluated in this article, or claim that may be made by its manufacturer, is not guaranteed or endorsed by the publisher.

